# Cholera in Cameroon, 2000-2012: Spatial and Temporal Analysis at the Operational (Health District) and Sub Climate Levels

**DOI:** 10.1371/journal.pntd.0005105

**Published:** 2016-11-17

**Authors:** Moise C. Ngwa, Song Liang, Ian T. Kracalik, Lillian Morris, Jason K. Blackburn, Leonard M. Mbam, Simon Franky Baonga Ba Pouth, Andrew Teboh, Yang Yang, Mouhaman Arabi, Jonathan D. Sugimoto, John Glenn Morris

**Affiliations:** 1 Emerging Pathogens Institute, University of Florida, Gainesville, Florida, United States of America; 2 Department of Environmental and Global Health, College of Public Health and Health Professions, University of Florida, Gainesville, Florida, United States of America; 3 Spatial Epidemiology and Ecology Research Laboratory, Department of Geography, College of Liberal Arts and Sciences, University of Florida, Gainesville, Florida, United States of America; 4 World Health Organization country office for The Republic of Cameroon, Yaoundé, Republic of Cameroon; 5 Cellule de Supervision, Suivi et Evaluation, Délégation Régionale de la Santé Publique du Centre, Yaoundé, Cameroun; 6 Field Epidemiology and Laboratory Training Program, University of Yaoundé, Yaoundé, Republic of Cameroon; 7 Department of Biostatistics, College of Public Health and Health Professions, University of Florida, Gainesville, Florida, United States of America; 8 Higher Institute of the Sahel, University of Maroua, Maroua, Republic of Cameroon; 9 Center for Inference and Dynamics of Infectious Diseases and Vaccine and Infectious Disease Division, Fred Hutchinson Cancer Research Center, Seattle Washington, United States of America; 10 Department of Medicine, College of Medicine, University of Florida, Gainesville, Florida, United States of America; Massachusetts General Hospital, UNITED STATES

## Abstract

**Introduction:**

Recurrent cholera outbreaks have been reported in Cameroon since 1971. However, case fatality ratios remain high, and we do not have an optimal understanding of the epidemiology of the disease, due in part to the diversity of Cameroon’s climate subzones and a lack of comprehensive data at the health district level.

**Methods/Findings:**

A unique health district level dataset of reported cholera case numbers and related deaths from 2000–2012, obtained from the Ministry of Public Health of Cameroon and World Health Organization (WHO) country office, served as the basis for the analysis. During this time period, 43,474 cholera cases were reported: 1748 were fatal (mean annual case fatality ratio of 7.9%), with an attack rate of 17.9 reported cases per 100,000 inhabitants per year. Outbreaks occurred in three waves during the 13-year time period, with the highest case fatality ratios at the beginning of each wave. Seasonal patterns of illness differed strikingly between climate subzones (Sudano-Sahelian, Tropical Humid, Guinea Equatorial, and Equatorial Monsoon). In the northern Sudano-Sahelian subzone, highest number of cases tended to occur during the rainy season (July-September). The southern Equatorial Monsoon subzone reported cases year-round, with the lowest numbers during peak rainfall (July-September). A spatial clustering analysis identified multiple clusters of high incidence health districts during 2010 and 2011, which were the 2 years with the highest annual attack rates. A spatiotemporal autoregressive Poisson regression model fit to the 2010–2011 data identified significant associations between the risk of transmission and several factors, including the presence of major waterbody or highway, as well as the average daily maximum temperature and the precipitation levels over the preceding two weeks. The direction and/or magnitude of these associations differed between climate subzones, which, in turn, differed from national estimates that ignored subzones differences in climate variables.

**Conclusions/Significance:**

The epidemiology of cholera in Cameroon differs substantially between climate subzones. Development of an optimal comprehensive country-wide control strategy for cholera requires an understanding of the impact of the natural and built environment on transmission patterns at the local level, particularly in the setting of ongoing climate change.

## Author Summary

Cholera was first reported in Cameroon in 1971. From 2000–2012, Cameroon reported on average 3,344.2 cases per year. When we divided the country into its four climate subzones (Sudano-Sahelian, Tropical Humid, Guinea Equatorial, and Equatorial Monsoon), there were very different patterns of spatial clustering of health districts with elevated attack rates, as well as differing sets of ecological determinants of cases counts. In the northern Sudano-Sahelian climate subzone, reported cases tended to occur between July and September, during the rainy season; whereas, the southern Equatorial Monsoon subzone reported cases year-round, with the lowest burden during the same rainy season. As cholera displays different epidemiological patterns by subzone, a single approach to controlling cholera for the whole nation does not appear to be viable. Additional prospective epidemiological studies are needed to further elucidate subzone-specific determinants of cholera burden, in order to provide sufficient evidence-based guidance for the formulation and assessment of regionally tailored intervention strategies.

## Introduction

Cholera is a severe acute watery diarrheal disease caused by toxigenic strains of *Vibrio cholerae*. The causative bacterium may be free-living in the environment, with environmental reservoirs well described in endemic regions in Asia [1]; the microorganism can live in both fresh- (inland lakes) [2, 3] and salt-water (coastal, estuarine areas) environments [1]. Illness tends to occur in seasonal patterns [4–6], with seasonality often associated with environmental parameters such as rainfall [7] and temperature [8, 9]. While the environmental link is important for maintaining the microorganism long-term (including time between epidemics), once introduced into human populations *V*. *cholerae* rapid (epidemic) transmission is associated with poor sanitation [10, 11], household contamination [12, 13], and contamination of food [14, 15] and potable water [12]. Epidemiologic studies in Africa have clearly documented the association between environmental exposures and occurrence of illness [13–15]; at the same time, there are also data suggesting that a great deal of the transmission in Africa is due to direct transmission among humans [16, 17]. Understanding these transmission patterns is a critical element in designing interventions as part of national and global cholera control programs.

Since 1817, seven cholera pandemics have spread from South and Southeast Asia to the rest of the world, and the seventh that began in 1961 in Indonesia is ongoing [18, 19]. During 2014, 190,549 cholera cases and 2,231 deaths (case fatality ratio [CFR] of 1.17%) were reported to the World Health Organization (WHO) by the public health authorities of 42 countries [20]. Yet, the actual disease burden is estimated to be much higher, in the range of 1.3 to 4.0 million cholera cases and 21,000–143,000 deaths per year worldwide [21]. Cholera is a threat to public health globally, but its burden is biased towards developing countries where poor water and sanitation provide added opportunities for transmission.

The current pandemic reached Africa in 1970, with the first cases reported in Guinea-Bissau [22] and Guinea Conakry [23]. Between 1980 and 2005, Africa accounted for the majority of the reported global burden of cholera [24]. From 2000 to 2014, African countries reported 2,139,424 cases of cholera, in comparison to the 186,401 from Asia; 754,694 from the Americas; 16,291 from Oceania; and the 417 from Europe [25]. The four countries around the Lake Chad Basin (Niger, Nigeria, Chad, and Cameroon) reported 62,762 cases in 2010; 65,401 in 2011; 6,784 in 2012; 7,215 in 2013; and 41,410 in 2014 [20, 26–29]. Of these four countries, Cameroon was responsible for 22,762 cases and 786 deaths (3.5% CFR) in 2011 [30]. In 2014, Cameroon was among the four countries in Africa with a CFR >5% [20], far exceeding the WHO target of less-than 1%.

In considering approaches to control cholera in Cameroon, there are clear knowledge gaps regarding the fine-scale spatial and temporal distributions of disease burden, as well as the human and environmental drivers of disease transmission in this setting. For evidence-based planning of interventions programs, an understanding of these factors is urgently needed. The current study uses a unique health district-level dataset to address these issues in the context of Cameroon.

## Methods

### Study Setting

**Fig 1:**
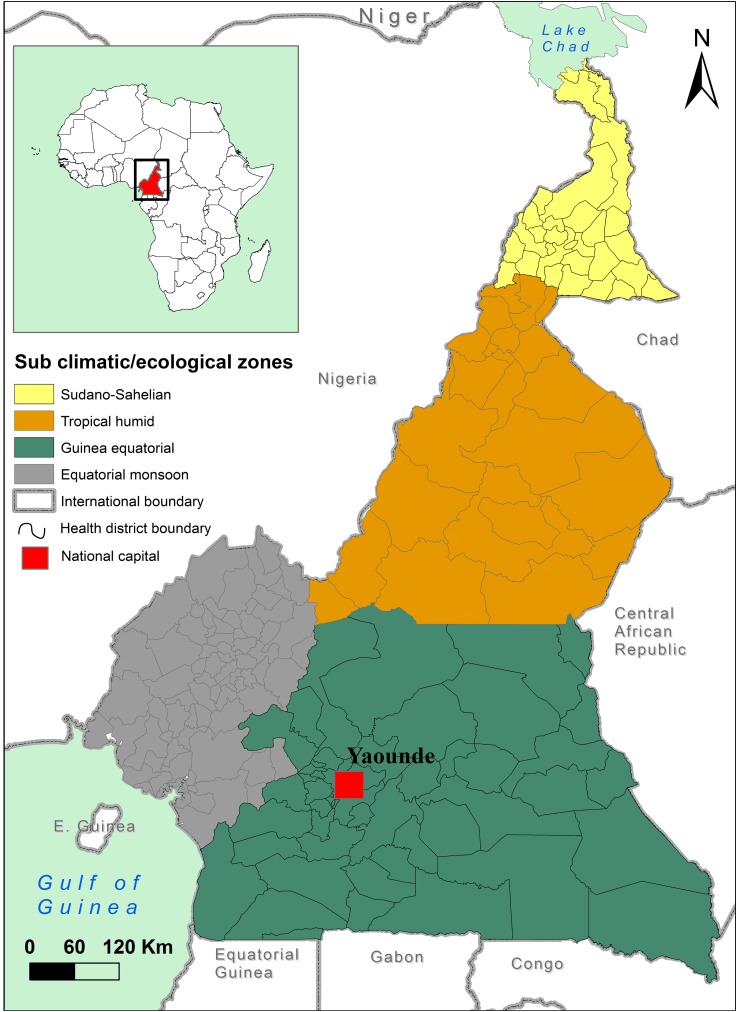
Neighboring countries and the four climate subzones of Cameroon (following Moloua and Lambi [32]). The capital Yaoundé has been added for orientation.

Cameroon is situated on the Atlantic Coast of central Africa (Fig 1), sharing borders with Nigeria (West), Chad (Northeast), and Central African Republic (East), Republic of the Congo, Gabon, and Equatorial Guinea (South for the latter three). The country is divided into 10 semi-autonomous regions, and 183 health districts, with an estimated population (July, 2015) of 23,739,218 [31]. Cameroon can be divided into four distinct climate subzones: the well-watered southern Equatorial Monsoon and Guinea Equatorial subzones transition through the Tropical Humid subzone in the middle of the country to the semi-arid Sudano-Sahelian subzone in the north (Fig 1) [32]. The second wettest place on earth (after Chirapunji, India) is located in the Equatorial Monsoon subzone, reporting an annual rainfall between 2000 and 10,000 mm and a mean yearly temperature of 26°C [32]. The Guinea Equatorial subzone has two rainy seasons with an annual average rainfall of 1500 to 2000 mm and an annual mean temperature of 25°C. In the Tropical Humid subzone, 900 to 1500 mm of rain are reported during a 4–5 month wet season, with an annual mean temperature of 28°C. The Sudano-Sahelian rainy season is shorter, with a reported 400 to 900 mm of rain and an annual mean temperature of 28°C.

### Data Collection

We obtained health-district level data on the number of cholera cases and deaths from the WHO country office for the period from 2000 to 2010 and from the Cameroon Ministry of Public Health (MoPH) for 2011 and 2012. Data for all years were collected through healthcare facility based passive surveillance, as previously described [33]. In brief, cholera data are collected at health facility and sent to health district office where data are compiled and sent to the Regional Public Health Delegation (RPHD) in paper format. RPHD de-identifies, digitizes, and sends the data to the MoPH via the internet and from there to the WHO [33]. The 2005 population size of each health-district was obtained from the most recent population and housing census in Cameroon [34]. Previously-published growth estimates [35] were used to estimate the average annual population for each health district for 2000–2004 and 2006–2010. For 2011 and 2012 the population estimates were extracted directly from the cholera surveillance data provided by MoPH. Use of these datasets was approved by the University of Florida IRB, and by the ethical committee in the Cameroon Ministry of Public Health.

We obtained coastline/shoreline and highway (trunk and primary roads) shapefiles from [36]; shapefiles for potential environmental reservoirs for cholera, including lakes and rivers, were obtained from DIVA-GIS [37]. Using ArcGIS Version 10.2 (ESRI, Redlands, CA, USA) and custom Python code, we generated a polygon shapefile from a paper map of health district boundaries. Polygon shapefiles for the national and regional boundaries were obtained from the spatial data accompanying ArcGIS 10.2. All spatially-referenced data was projected into the Universal Transverse Mercator, Zone 33N, coordinate system.

Daily rainfall levels (mm) were obtained in the form of 0.1°-resolution raster files (coverage included the whole continent) from the Famine Early Warning System Network [38], which is itself based upon the African Rainfall Climatology (version 2) dataset [39]. The African Flood and Drought Monitor (based upon the Global Forecasting System Analysis Dataset) [40] provided daily maximum temperature data in the form of 0.25°-resolution NetCDF files (coverage included the whole continent). After converting the NetCDF files to a raster format, the temperature and precipitation files were projected and clipped to the boundaries of Cameroon. The weekly averages for the daily rainfall and maximum temperature were calculated for each pixel and then further averaged across the pixels located within a health district.

### Descriptive and Seasonal Variation Analysis

To visualize spatial and temporal trends in the health-district-level crude attack rates (AR’s) of reported cholera cases per 100,000 inhabitants (number of reported cholera cases during a week / annual population estimate), annual choropleth maps were produced for 2000 to 2012. To address the concern that spatial trends in the average annual crude AR were solely statistical artifacts of the data [41], Empirical Bayes Smoothed (EBS) AR’s were estimated using GeoDa (version 1.6.6.1) [42] and compared to the corresponding crude ARs via box plots, using SAS version 9.4 [43], and via choropleth maps (ArcGIS 10.2). As there were no meaningful differences between the crude and EBS AR’s (S1 Fig), we proceeded with using the crude AR’s (henceforth referred to as solely AR’s) for the rest of our analyses.

### Spatial Clustering Analysis

To describe the spatial distribution of reported cholera cases across Cameroon, we conducted an analysis to describe the spatial clustering of weekly AR’s at the health-district level. We chose to restrict our analysis to the years of the study period with the most reported cholera cases (2010–2011). Spatial clusters of health districts with high annual AR’s were identified using SaTScan v.9.4.1 through a retrospective analysis of the data for this two-year period using a Poisson discrete spatial scan statistic [44], with a 50% spatial window and default values used for all other settings. To assess the sensitivity of our clustering analysis to the size of the spatial window, this parameter was lowered to 25%, with no appreciable change in the nature of the identified spatial clusters (S2 Fig).

### Spatiotemporal Autoregressive Poisson Regression Analysis

Another of our objectives was to explore the associations between environmental and structural factors and the burden of reported cholera cases at the health district level, with the aim of using this information to inform the planning of cholera control programs. Changes in temperature [45] and precipitation [13, 15, 45] levels have been previously associated with the probability of subsequent cholera outbreaks. Local geographic features, such as highways [2], inland waterbodies (lakes/rivers) [2, 46], and coastline/shoreline [5, 47] have also been associated with elevated disease frequency.

We considered the mean daily maximum temperature (degrees Celsius) and the mean daily precipitation level (mm) over the preceding two weeks as potential environmental predictors of the reported number of cases during each week under study. We [48, 49] and others [50–52] have previously demonstrated a lag in the effects of climatic events on cholera case occurrence, particularly when transmission of *V*. *cholerae* O1 infections are primarily associated with exposure to environmental reservoirs and/or contaminated water sources.

To reduce the potential for model instability related to sparse disease burden data, we elected to discretize our continuous mean daily maximum temperature and mean daily precipitation level data into low (reference), medium, and high categories. The cut-off values delineating categories were chosen based upon 33rd and 67th percentiles (see Table 1 for specific cut-off values). Indicator variables were generated for the presence/absence (1 vs. 0) of each of highways and inland-waterbodies/coastline/shoreline.

**Table 1:** Incidence Rate Ratio (IRR) estimates from two multivariable spatiotemporal autoregressive Poisson model fits to reported cholera case counts for the health districts of Cameroon during 2010–2011.

Parameters	Climate Subzone Specific Model				National Model (NM) IRR^a^ (95% CI)
	Sudano-Sahelian (SS) IRR^a^ (95% CI)	Tropical Humid (TM) IRR^a^ (95% CI)	Guinea Equatorial (GE) IRR^a^ (95% CI)	Equatorial Monsoon (EM) IRR^a^ (95% CI)	
Hazard of reported cholera associated with exposure to the preceding week’s (*t*-1) cases for the same health district, (NM, λx10^-6^; All others, λ_Z_x10^-6^)	2.64 (2.04–3.40)	3.43 (2.13–5.54)	1.64 (1.41–1.90)	5.05 (4.55–5.60)	3.03 (2.86–3.21)
Mean of the maximum daily temperature (°C)^b^					
Medium (M) versus Low (L)	1.09 (1.04–1.15)	0.85 (0.78–0.92)	1.56 (1.42–1.71)	0.87 (0.82–0.93)	0.93 (0.90–0.96)
High (H) versus Low (L)	1.87 (1.71–2.05)	0.89 (0.80–0.99)	1.72 (1.51–1.96)	0.46 (0.43–0.49)	1.42 (1.38–1.46)
Mean daily precipitation (mm)^b^					
Medium (M) versus Low (L)	1.33 (1.02–1.73)	0.81 (0.67–0.98)	1.19 (1.03–1.38)	1.11 (1.05–1.17)	1.23 (1.18–1.28)
High (H) versus Low (L)	1.99 (1.54–2.56)	0.62 (0.51–0.75)	1.30 (1.12–1.51)	0.96 (0.91–1.01)	1.30 (1.24–1.36)
Presence of a major body of water (inland or ocean)	1.32 (1.22–1.43)	4.02 (2.56–6.33)	1.48 (1.20–1.84)	1.11 (1.06–1.16)	1.11 (1.07–1.13)
Presence of a Highway	0.97 (0.93–1.02)	2.48 (1.59–3.87)	1.14 (1.05–1.25)	0.97 (0.90–1.05)	0.88 (0.86–0.91)

CI, confidence interval; IRR, incidence rate ratio, represents the incidence rate among the population exposed to cholera risk factor(s), divided by the incidence rate among the unexposed. As measure of the strength of association, an IRR = 1 suggests no difference in risk among exposed and unexposed groups, IRR > 1 suggests increased risk in the exposed group, and IRR < 1 suggests decrease risk in the exposed group.

^**a**^ With the exception of *λ*, all estimates represent IRR’s. Note: *λ* for the NM differs from ***λ***_***z***_ for the climate subzones because hazards of transmissions are different in the different zones across Cameroon.

^**b**^ Category definitions for Temperature [**Subzone-specific model**: SS (L < 34.36, M = 34.37–37.02, H > 37.02); TH (L < 32.17, M = 32.18–35.65, H > 35.65); GE (L < 29.63, M = 29.64–30.87, H > 30.87); EM (L < 27.87, M = 27.88–30.28, H > 30.28); **National model**: L < 29.46, M = 29.47–32.03, H > 32.03], and Precipitation [**Subzone-specific models**: SS (L < 0.34, M = 0.35–22.07, H > 22.07); TH (L < 3.59, M = 3.60–33.64, H > 33.64); GE (L < 18.25, M = 18.26–33.59, H > 33.59); EM (L < 22.39, M = 22.40–44.79, H > 44.79); **National model**: L < 14.28, M = 14.29–36.31, H > 36.31].

### Poisson Regression at the National and Climate Subzones Scales

In keeping with our focus on exploring the potential importance of subnational variability in the epidemiology of cholera for informing effective control strategies and programs, we fit a regional (climate subzone) and a national version of a multivariable spatiotemporal autoregressive Poisson regression model to the weekly number of cholera cases reported in each health district during 2010 and 2011. The first model (subzone-specific) estimated separate incidence rate ratio (IRR) values for each climate subzone, but inferred nationwide versions of the two key IRRs related to the spatial and temporal influences of recent cholera burden levels on the number of reported cases in each health district; here IRR is the measure of the strength of association among the population exposed to cholera risk factors and those unexposed. The second model made the more restrictive assumption that there exists a single set of parameters shared by all health districts, independent of their climate subzone. We chose to aggregate health districts by climate subzone as these subzones differ substantially in the levels and temporal patterns of temperature and precipitation (an approach advocated by [53]).

Our cholera frequency data consists of the number of healthcare-ascertained cholera cases, *Y*_*i*_(*t*), reported in health district *i* during each epidemiologic week *t* of 2010 and 2011 (referenced to the first calendar week of 2010). The number of individuals living in *i* during *t*, *N*_*i*_(*t*), is assumed to remain constant throughout a calendar year and to be equal to the annual average population size estimate. For the more general subzone-specific model, the distribution of *Y*_*i*_(*t*) is assumed to be Poisson with mean

$$\mu_{i}\left(t\right)=\lambda_{0}+\lambda_{z}e^{\beta_{\text{z}}^{\text{T}}{\text{X}}_{\text{i}}\left(\text{t}-\text{l}\right)}\left[Y_{i}\left(t-1\right)+\varphi Y_{i}\left(t-2\right)+\theta \sum_{j\neq i}\omega_{ji}\left(Y_{j}\left(t-1\right)+\varphi Y_{j}\left(t-2\right)\right)\right]$$

and an offset, *Ni*(*t*), where *λ*_0_ is the hazard of reported cholera cases associated with environmental exposure, and *λ*_*z*_ represents the climate subzone (*z*) specific hazard of reported cholera cases associated with exposure to cholera cases reported in the same health district during week *t* − 1. The parameter *φ* represents the marginal relative risk (across all health districts) comparing transmission associated with exposure in the same health district during weeks *t* − 2 versus *t* − 1. In contrast, *θ* characterizes the marginal relative risk (across all health districts) for transmission associated with exposure in another health district during weeks *t* − 2 or *t* − 1 compared to exposure in one’s own health district during week *t* − 1. To account for the likely dependence of the probability of being exposed to cholera in another health district on its geographic distance from a person’s home district, deterministic importance weights were assigned to all neighboring health districts *j* of *i* using a modified gravity, where $\omega_{ji}=\frac{1/d_{ji}}{\sum_{k\neq j}1/d_{jk}}$, with *d*_*ji*_ representing the Euclidean distance between the geographic centroids of *i* and *j* if ≤ 500 kilometers or infinity otherwise. The vector *X*_*i*_(*t* − *l*) contains the environmental and structural covariate values associated with health district *i* during week *t* − *l*, where *l* is the pre-specified time lag, and the coefficients for their effects are estimated by the climate subzone specific vector *β*_*z*_. The incidence rate ratios for the effects of the environmental and structural covariates are given by $e^{\beta_{z}}$. To avoid non-identifiability issue, the environmental hazard *λ*_0_ is not adjusted for covariates. The form of *μ*_*i*_(*t*) for the national model is similar, with the exception that *λ*_*z*_ and *β*_*z*_ are replaced with the national-level parameters *λ* and *β*, respectively. Both models are similar to the regression models of Paul and Held (for example, [54]), with the difference that our autoregressive component incorporates more than one time step.

Prior to fitting our models, the level of collinearity between each pair of the environmental and structural covariates was examined. Model fitting was conducted using the R statistical computing environment (version 3.2.5) [55]. The fit of the model to the data was assessed qualitatively using plots of the predicted and observed cases counts.

## Results

### Descriptive Statistical and Spatial Mapping Analysis

**Fig 2:**
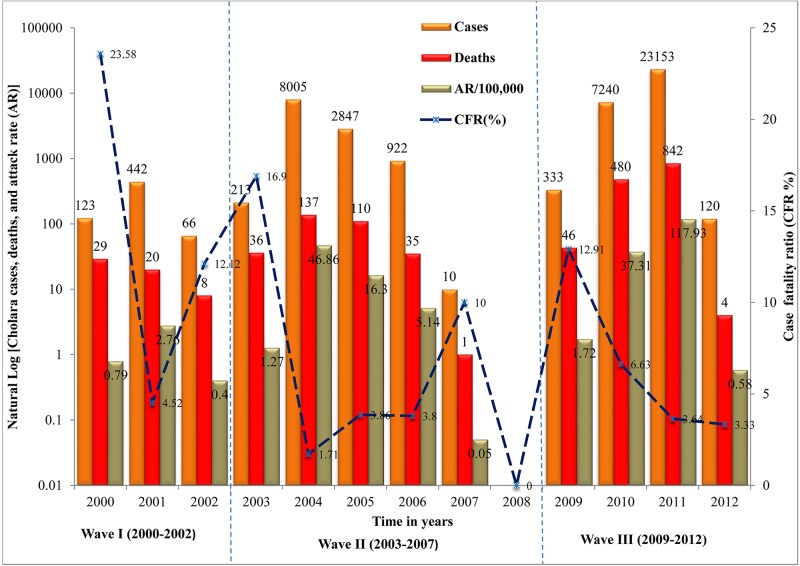
Yearly occurrence of cholera outbreaks in Cameroon from 2000 to 2012. The vertical axis for the cases (gold color), deaths (red color), and attack rates (AR) are in log scale. Blue dashed line represents case fatality ratio (CFR).

In the 13-year period under study, 43,474 cholera cases were reported, of which 1748 were fatal (CFR of 4%). The mean annual attack rate was 17.78 cases per 100,000 individuals, with a mortality rate of 0.71 deaths per 100,000 population. Between 2000 and 2012, outbreaks occurred in three waves (Fig 2). Wave I appeared between 2000 and 2002 and peaked in 2001 at an AR of 4.52 per 100,000 inhabitants (2.76% CFR). Wave II started in 2003 with an initial AR of 1.27 per 100,000 individuals and peaked in 2004 at an AR of 46.86 per 100,000 (CFR of 1.71%). A brief intermission was observed in 2008 before Wave III that began in 2009 with an AR of 1.72 per 100,000 and a CFR of 12.91%. The cholera AR increased steadily throughout the third wave to a peak of 117.93 per 100,000 in 2011 (3.64% CFR). The CFR was consistently highest at the start of each wave and lowest at its peak.

**Fig 3:**
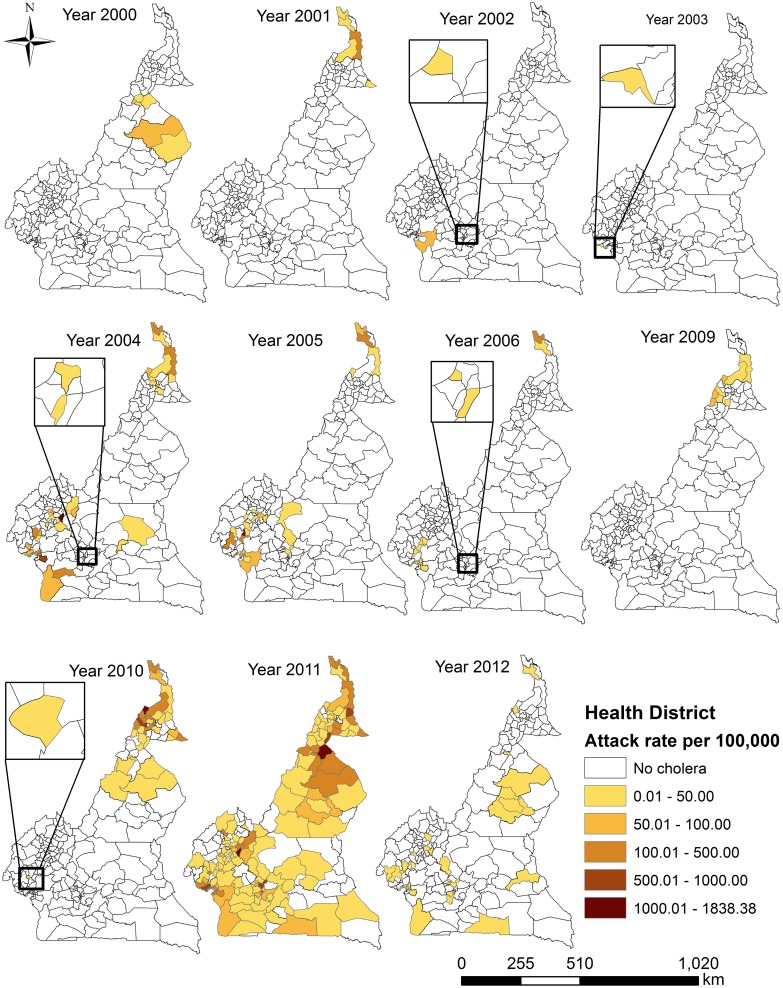
Variation of crude health district attack rates from 2000–2012. Maps show outbreaks occurring either in the north or south or both, with a north-south divide. The north-south divide narrowed for the first time in 2011, but four districts remained free of reported cholera cases. There were no data for 2007 at district level, and there was no cholera reported in 2008.

The distribution of health districts with elevated burdens of reported cholera cases demonstrated substantial spatial heterogeneity from year to year (Fig 3). Between 2000–2003 and 2009–2010, the majority of the burden of reported cases was concentrated in either the northern or the southern portions of Cameroon, whereas, the other years of our study period saw the burden spread throughout the northern and southern sections of the country. The widest geographic distribution of cases occurred in 2011, but four health districts in the middle of the country remained free of reported cholera cases.

### Seasonal Variation of Cholera at the Level of the Climate Subzone

**Fig 4:**
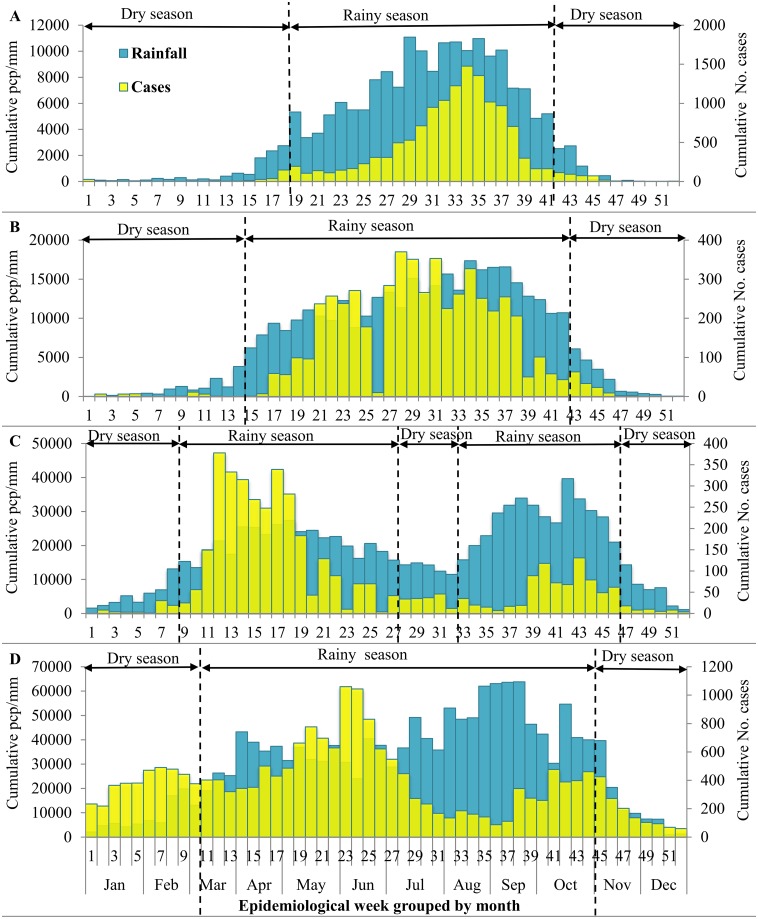
Seasonal variation of reported cholera cases in Cameroon. Graphs show cholera cases (yellow color) and precipitation (pcp) (blue color) by week and month in the Sudano-Sahelian (A), Tropical Humid (B), Guinea Equatorial (C), and Equatorial Monsoon (D) climate subzones of Cameroon, 2000–2012. In the Sudano-Sahelian, Tropical Humid, and Guinea Equatorial subzones cholera is predominantly in the rainy season, with cases appearing year-round in the Equatorial Monsoon subzone.

Between 2000 and 2012, the health districts of the Sudano-Sahelian and Tropical Humid subzones demonstrated average annual temporal patterns of case burden consistent with a single, epidemic season that overlapped almost exactly with the rainy season (April/May to October), with peaks in July/August (Fig 4A and 4B). On average between 2000 and 2012, elevated burdens of reported cholera cases in the Guinea Equatorial climate subzone were observed during both of the rainy seasons (March-June and August-November), with the peak burdens in April and October (Fig 4C). Significant numbers of cholera cases were reported year-round for health districts in the Equatorial Monsoon subzone, with lower burdens reported during the peak rainfall in August and September (Fig 4D). For 2010 and 2011, Fig 5 presents subzone-specific temporal trends in the reported cholera case counts and in the weekly means for daily maximum temperature and precipitation.

**Fig 5:**
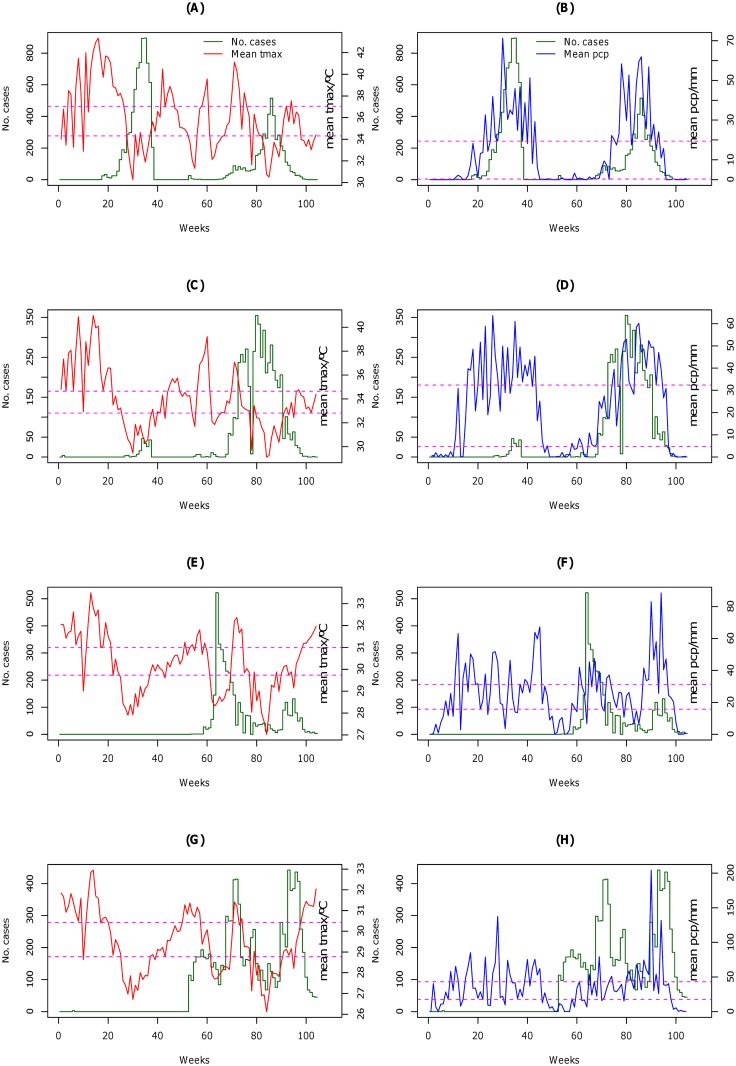
Temporal trends in reported cholera cases for 2010 and 2011. The figure shows mean maximum temperature (tmax), and precipitation (pcp) in the Sudano-Sahelian (A and B), Tropical Humid (C and D), Guinea Equatorial (E and F), and Equatorial Monsoon (G and H) climate subzones of Cameroon. The primary y-axes are cases (dark green), secondary y-axes, mean tmax (red) and pcp (blue), respectively. Cut-off points (dashed purple line) were based on 33rd and 67th percentiles.

### Spatial Clustering of High Incidence Health Districts during 2010 and 2011

**Fig 6:**
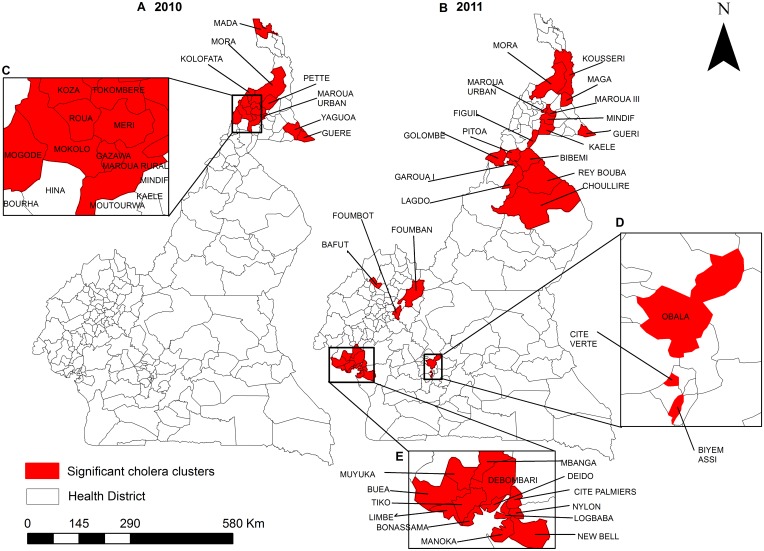
Spatial clusters of cholera in Cameroon at the national level for 2010 (A) and 2011 (B). Cluster analysis used default settings (50% spatial window). Districts clustered disproportionately in all four climate subzones. Red color show statistically significant clusters. Inserts C, D, and E show a better view of clusters.

Statistically significant spatial clusters of health districts with high AR’s for reported cholera cases were identified in the Sudano-Sahelian subzone during 2010, primarily concentrated in the mountainous border region with Nigeria (Fig 6A). We also identified clusters bordering Lake Chad and in the eastern Logon-Chari floodplains. In 2011, clusters in the Sudano-Sahelian zone shifted from the mountainous highlands to the eastern floodplains (Fig 6B). Furthermore, we identified additional significant spatial clusters in the northeastern section of the Tropical Humid subzone during 2011. Elevated cholera burden clustered significantly among the urban health districts of the national capital, Yaoundé, located in the Guinea Equatorial subzone, as well as among Equatorial Monsoon subzone health districts along the Atlantic coast and in its northwestern section (Fig 6B). With the exception of the Yaoundé in 2011, no statistically significant spatial clusters were detected in the middle section of the country during either year, reflecting the apparent north versus south divide of cholera occurrence.

### Poisson Regression at the National and Climate Subzones Scales

Most covariates were found to be significantly associated with cholera transmission at the health district level (Table 1). The trends in the estimated effects (IRRs) of the average daily maximum temperature and the average daily precipitation differed by climate subzone. For the Sudano-Sahelian and Guinea Equatorial subzones, increasing levels of temperature and precipitation were each associated with incrementally higher intensities of transmission. In contrast, the health districts of the Tropical Humid and Equatorial Monsoon subzones exhibited a negative association between temperature and precipitation levels and transmission intensity, with the exception of precipitation for the Equatorial Monsson subzone where the IRR for the medium level precipitation setting was significantly greater than 1.0.

**Fig 7:**
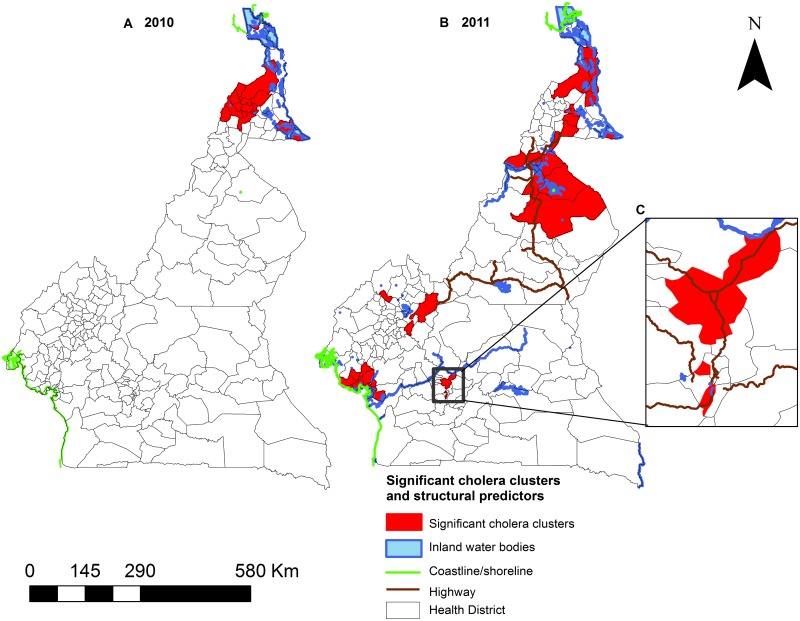
Spatial clusters and statistically significant risk factors. In the Sudano-Sahelian, cholera clustered around inland waterbodies. In the Tropical-Humid and Guinea Equatorial, disease clustered around both inland waterbodies and highways. In the Equatorial Monsoon disease clusters appeared predominantly in health districts along the coastline. Insert gives a detail view of cluster and the structural/environmental predictors around the capital Yaoundé.

The estimated effects of the presence of a major body of water and of a highway (Fig 7) in a health district differed between climate subzones. The presence of a major waterbody was associated with elevated transmission intensity, where the estimated IRR ranged from 1.11 (95% CI: 1.05–1.16) in the Equatorial Monsoon subzone to 4.02 (95% CI: 2.56–6.33) in the Tropical Humid health districts. Highways were estimated to elevate transmission intensity of cholera in only the Tropical Humid and Guinea Equatorial subzones.

The national model estimated IRR’s for the medium and high temperature categories that were slightly lower than and substantially higher than 1.0, respectively. In contrast, the estimates for the effects of precipitation from the national model followed a positive trend similar to the subzone specific estimates from the Sudano-Sahelian and Guinea Equatorial subzones. The presence of major waterbodies and highways (Fig 7) were estimated to slightly increase and decrease the intensity of cholera transmission by 15%, respectively.

**Fig 8:**
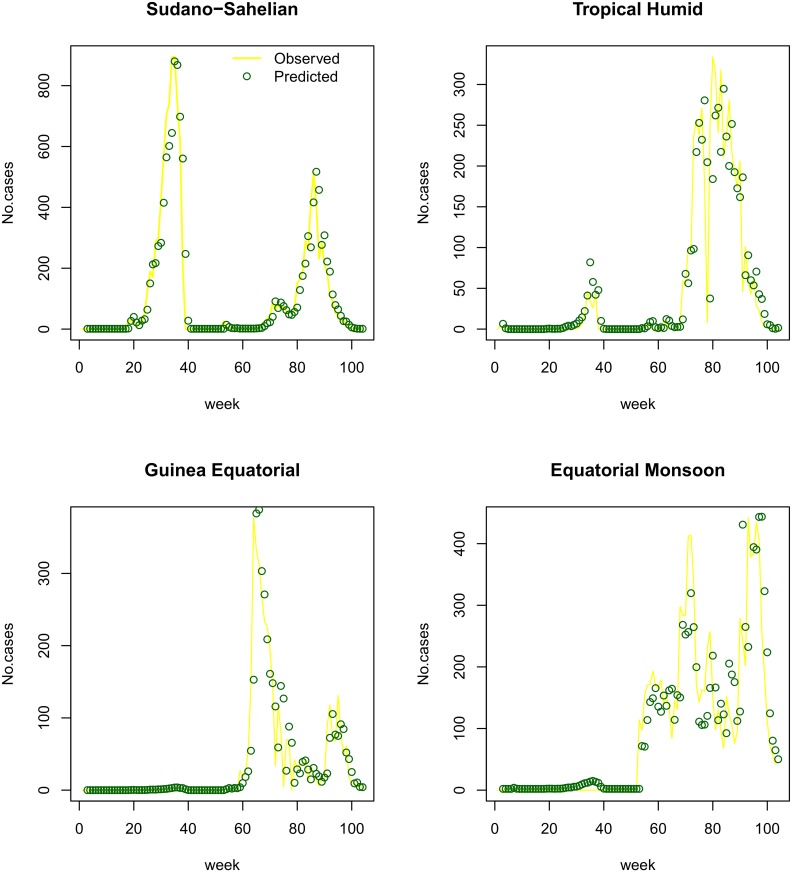
Climate subzone level model goodness of fit for Cameroon in 2010–2011. Predicted Weekly case numbers (green dots) versus observed data (yellow line) show goodness of fit in Sudano-Sahelian, Tropical Humid, Guinea Equatorial, and Equatorial Monsoon climate subzones.

**Fig 9:**
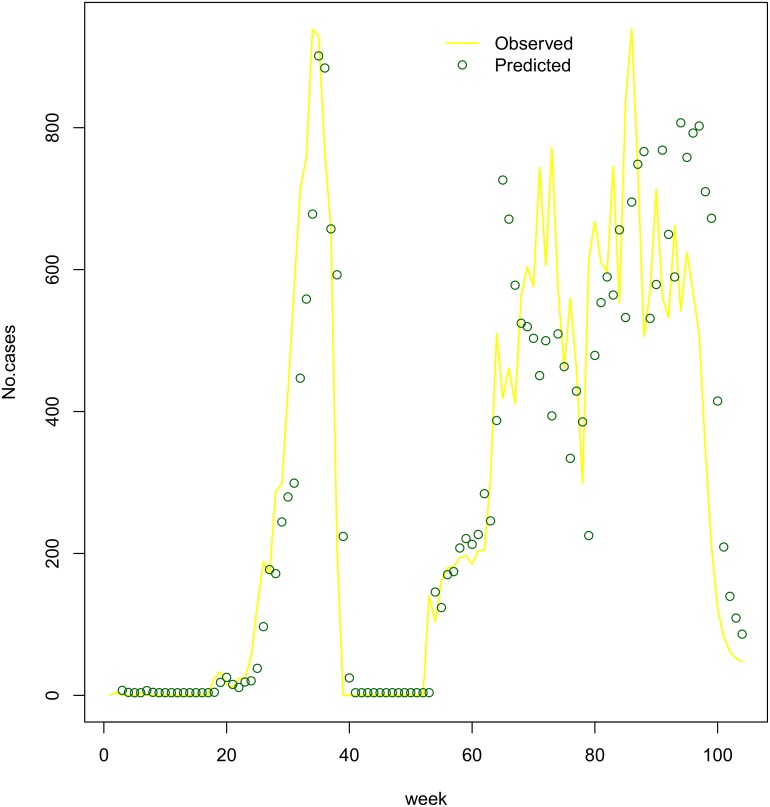
National level model goodness of fit for Cameroon in 2010–2011. Predicted Weekly case numbers (green dots) versus observed data (yellow line).

Estimates of *λ*_*z*_ and *λ* were consistent in scale between the two regression models. The estimate of IRR for the temporal autocorrelation effect *φ* was 0.15 (95% CI: 0.14–0.17) for the subzone-specific model and 0.13 (95% CI: 0.12–0.15) for the national model. For the subzone-specific and national models, the IRR’s for the spatial autocorrelation effect *θ* were estimated at 0.16 (95% CI: 0.15–0.17) and 0.18 (95% CI: 0.17–0.19), respectively. Both models appeared to qualitatively fit the data well (Figs 8 and 9).

## Discussion

This study analyzed cholera disease frequencies, distribution patterns, and explored risk factors that might have been associated with disease transmission in Cameroon from 2000 to 2012. In this time period, annual epidemics waxed and waned in three waves with a 3-to-5 year life cycle, as has been identified in India (3-year cycles) [56] and Bangladesh (3-to-6 year cycles) [6]. The cholera data used in the analyses encompass hospital discharge data from a passive surveillance system [33], which almost certainly under represent the true burden of cholera infection in each health district. However, the true burden may also be over-reported since other infections may also cause severe watery diarrhea. Nonetheless, the data provide a unique opportunity to further explore factors linked with transmission risk, and to do so at a climate subzone level.

From a public health stand point, the observed variation in the CFR (highest at the onset of each wave of illness, lowest at the peak) highlights the need for early detection of outbreaks via enhanced public health surveillance and a rapid response at the health district level; a strategy that is already stipulated by the Integrated Disease Surveillance and Response strategy [57]. However, outbreak response at the district level in Cameroon is likely to present significant logistical challenges [33]. The results of our study suggest that stockpiles will be needed for sufficient supplies to respond to a 3-to-5 year long cholera outbreak. The logistics need to be worked out in advance for ensuring an efficient flow of resources from the manufacturer to the end user, *i*.*e*., the district level outbreak response team. The need for such advanced planning is illustrated by anecdotal reports that during the 2010–2011 outbreaks, some health facilities ran out of supplies (personal communication in 2013 with Dr. Ellen Einterz, Medical doctor at the Kolofata health district hospital, Far North). The tangible consequences of inadequate levels of supplies and available personnel to respond to a cholera outbreak can be seen in a 2009 study in the Far North Region of Cameroon, which found that reluctance to seek care for cholera, the distance to the nearest healthcare facility, and the less than adequate availability of oral rehydration salts and antibiotics at healthcare facilities were significantly associated with elevated risk of the cholera-associated death [58].

The WHO estimates that in 2012, 74% (urban 95%, rural 52%) of Cameroonians had access to improved water while 45% (urban 62%, rural 27%) used proper sanitation [59]. To reduce cholera AR’s from the high levels found in this analysis, improvements to water and sanitation system deficiencies are urgently needed. While investments in water, sanitation, and behavior change are long-term measures, an investment in cholera vaccines [20] would be an appropriate short-term measure to mitigate high attack rates in the interim. To the credit of public health programs in Cameroon, we observed a decreasing trend in CFR over this 13-year period. Yet, the current CFR still far exceeds the <1% WHO standard. This underscores the need both for public education, to encourage patients to rapidly seek medical attention, and for improvements in health facilities in terms of personnel training to bring the CFR below 1% for future outbreaks. Nevertheless, a focus on achieving a < 1% CFR goal implies focusing on disease response rather than prevention [60], which is in contrast to the current WHO recommendation to shift to a disease prevention mindset [60]. After all, proactive disease prevention is, actually, what set the worlds of public health and biomedical sciences apart.

Similar to results from other settings [13, 15, 45], our analysis identified recent trends in temperature and precipitation as being significantly associated with reported cholera cases, and we further demonstrated that the effects of these environmental covariates differed by climate subzone. As documented in other countries such as Chad [61], Côte d’Ivoire [62], Angola [63], and Zanzibar [64], cholera in the Sudano-Sahelian, Tropical Humid, and Guinea Equatorial climate subzones seem to occur in the rainy season (Fig 4A and 4C); cholera seasonal variation in the latter subzone mimics the double maxima of rainfall. In contrast, a significant number of cases are reported year-round in the Equatorial Monsoon subzone (Fig 4D); the literature has documented, for this subzone, the onset of cholera in the dry season and its retreat with the coming of the heavy rains [65].

From 2000 to 2012, there was a clear tendency for health districts reporting cholera to cluster in the northern and southern portions of Cameroon (Fig 3). This general spatial pattern was confirmed by our spatial clustering analysis of the data from 2010 and 2011. Considered in conjunction with the elevated hazard of cholera cases associated with the presence of inland waterbodies (Lake Chad in the North) and coastline (Atlantic Ocean in the South), these observations are consistent with previously-described associations between proximity to coastal and inland waterbody environments and the occurrence of cholera outbreaks in sub-Saharan Africa [66, 67]. In all four-climate subzones, the presence of inland waterbody or coastline is associated with elevated burdens of reported cholera cases. For the Tropical Humid and Guinea Equatorial subzones, the presence of a highway is significantly associated with elevated cholera burdens. Both of these results are consistent with findings from the African Great Lakes Region [2, 3], and they are consistent with the idea that the waterbodies acted as environmental reservoirs, with highways facilitating spread through population movements [67]. The apparent dependence of disease burden on the movement of individuals via highways is consistent with the results of an earlier study of the phylogeography of *V*. *cholerae* isolates from human sources collected during the 2010–2011 epidemic in the Equatorial Monsoon subzone, which did not support the hypothesis that local transmission was primarily environmental-to-human in nature [68].

Spatial and temporal distributions of cholera burden and the effects of predictors varied substantially across Cameroon. It is evident from these results that the epidemiology of cholera differs substantially between climate subzones, and it is likely to exhibit additional microspatial variation within each subzone. The substantial spatial variation in the nature of the epidemiology of cholera across Cameroon strongly suggests that a single ‘one-size-fits-all’ national approach to prevention and control might not be optimal. A comparison of the epidemiologies of cholera inferred by our two Poisson models illustrates how different conclusions about the importance of key covariates could be drawn when examining national versus subnational data, potentially leading to different approaches to delivery of prevention and control strategies. Instead, there is a need to develop regionally or locally targeted prevention and mitigation strategies and plans. Unfortunately, resources (human and physical) for cholera control and prevention may be woefully scarce and unable to adequately deal with the complexity of the epidemiology of cholera in Cameroon.

This study has important limitations. The cholera data used in the analyses encompass hospital discharge data from a passive surveillance system [33]. Therefore, not only was the data not collected for the purpose for which it has been used in this study but also under/over represent the true burden of cholera because patients who did not visit a health facility were not part of this dataset. On the other hand, some of the acute watery diarrheas may not have been cholera. Thus, selection bias and unrepresentative sample issues cannot be ruled out. The lack of population structure data also restricted our ability to use age adjusted district attack rates. Although the Poisson model in this analysis has the capability to model trend and seasonality, the latter aspects were not considered in this analysis. Including them would have significantly enriched this study, and their inclusion would be an important addition to future work.

An earlier study in Cameroon called for a systematic assessment of cholera risk factors across Cameroon [69]. We believe that our study provides an important step towards cholera risk factor identification in Cameroon, with a particular emphasis on understanding geographic heterogeneity in these effects. However, better surveillance systems [33] and more detailed epidemiologic studies of transmission routes and identification of climate subzone-specific risks factors are urgently needed to provide the data required to optimize the delivery of prevention and control interventions at the local level.

## Supporting Information

S1 Fig Comparative boxplots for Empirical Bayes Smoothed and unsmoothed crude attack rates per 100000 population. Plots show no appreciable differences between Smoothed (s) and unsmoothed crude (un) rates; and thus, crude attack rates were used in analysis.
(TIF)

S2 Fig Spatial clusters of health districts with high annual AR’s for 2010–2011 at 25% spatial window. Figure assess the sensitivity of clustering analysis to the size of the spatial window, this parameter was lowered from 50% (default) to 25%, with no appreciable change in the nature of the identified spatial clusters.
(TIF)
