# Supplementary figures and images for: Cholera in Cameroon, 2000-2012: Spatial and Temporal Analysis at the Operational (Health District) and Sub Climate Levels
Source: PLoS Negl Trop Dis. 2016 Nov 17;10(11):e0005105. doi: 10.1371/journal.pntd.0005105 (PMC5113893; doi:10.1371/journal.pntd.0005105)

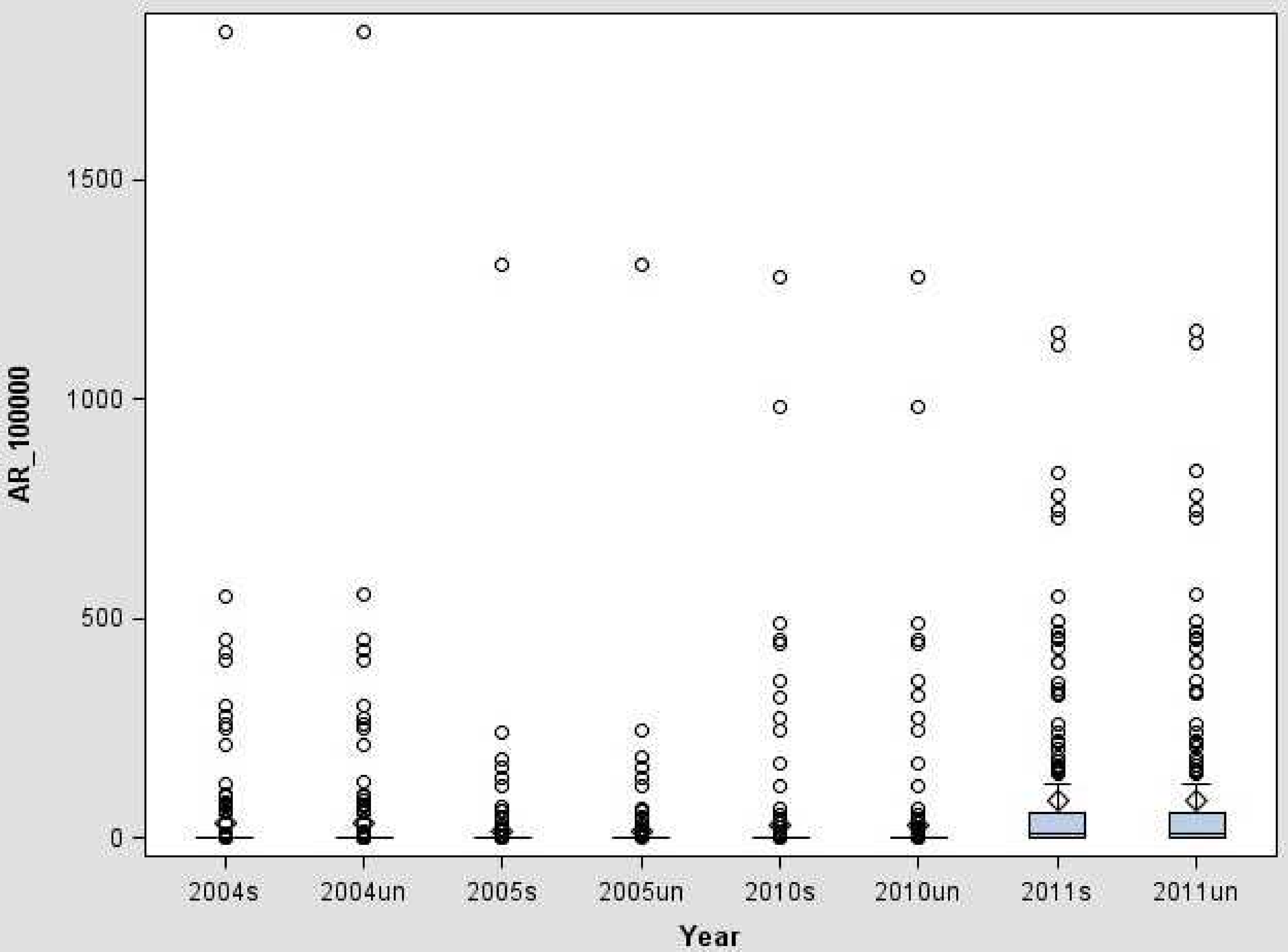

Supplement: S1 Fig — Plots show no appreciable differences between Smoothed (s) and unsmoothed crude (un) rates; and thus, crude attack rates were used in analysis. (TIF) [file pntd.0005105.s001.tif]

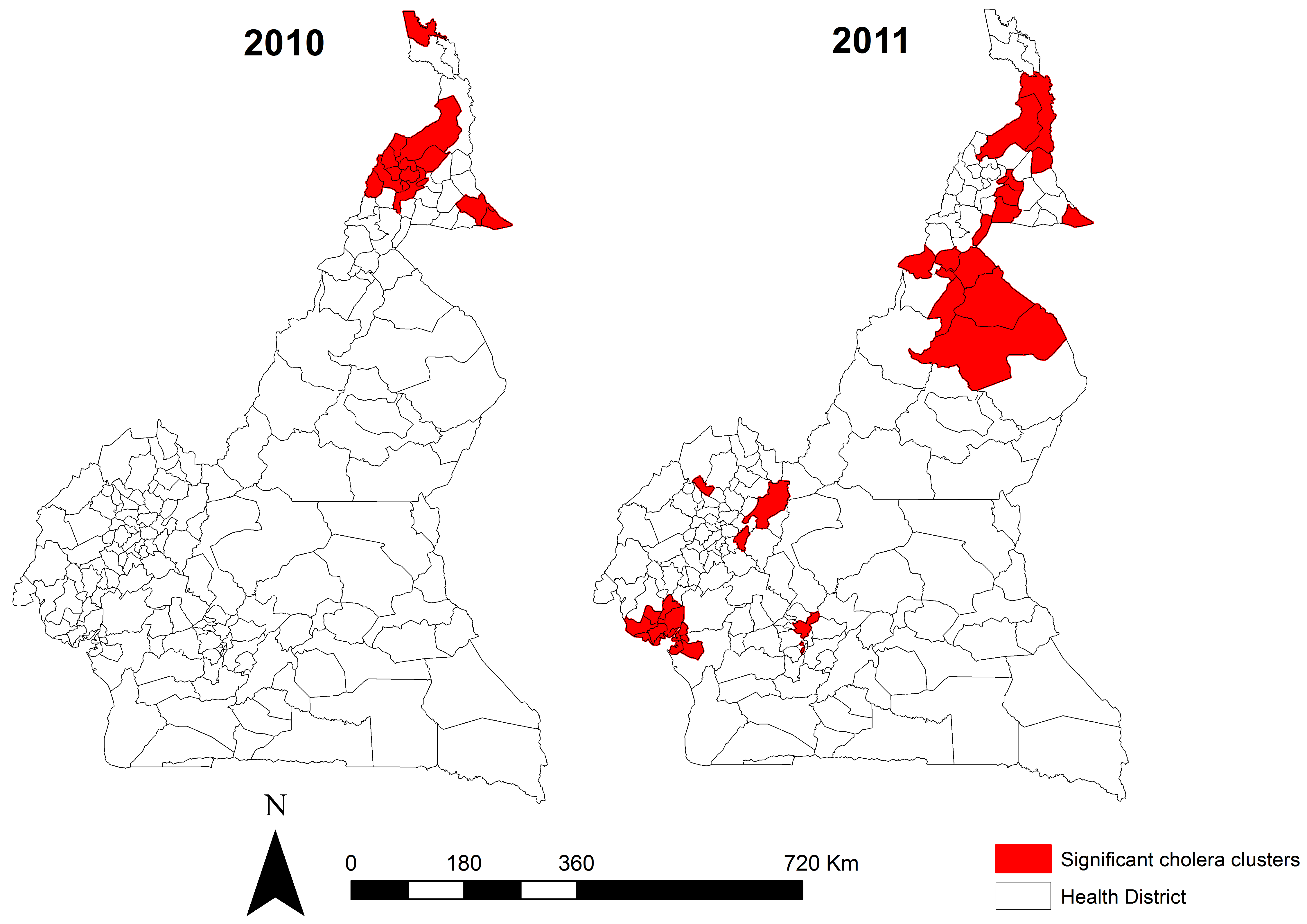

Supplement: S2 Fig — Figure assess the sensitivity of clustering analysis to the size of the spatial window, this parameter was lowered from 50% (default) to 25%, with no appreciable change in the nature of the identified spatial clusters. (TIF) [file pntd.0005105.s002.tif]
